# Correlation of transforming growth factor-β1 and tumour necrosis factor levels with left ventricular function in Chagas disease

**DOI:** 10.1590/0074-02760170440

**Published:** 2018-02-19

**Authors:** Eduardo OV Curvo, Roberto R Ferreira, Fabiana S Madeira, Gabriel F Alves, Mayara C Chambela, Veronica G Mendes, Luiz Henrique C Sangenis, Mariana C Waghabi, Roberto M Saraiva

**Affiliations:** 1 Fundação Oswaldo Cruz-Fiocruz, Instituto Nacional de Infectologia Evandro Chagas, Rio de Janeiro, RJ, Brasil; 2 Fundação Oswaldo Cruz-Fiocruz, Instituto Oswaldo Cruz, Rio de Janeiro, RJ, Brasil

**Keywords:** Chagas disease, tumour necrosis factor, transforming growth factor beta 1, brain natriuretic peptide, echocardiography

## Abstract

**BACKGROUND:**

Transforming growth factor β1 (TGF-β1) and tumour necrosis factor (TNF) have been implicated in Chagas disease pathophysiology and may correlate with left ventricular (LV) function.

**OBJECTIVES:**

We determined whether TGF-β1 and TNF serum levels correlate with LV systolic and diastolic functions and brain natriuretic peptide (BNP) serum levels in chronic Chagas disease.

**METHODS:**

This cross-sectional study included 152 patients with Chagas disease (43% men; 57 ± 12 years old), classified as 53 patients with indeterminate form and 99 patients with cardiac form (stage A: 24, stage B: 25, stage C: 44, stage D: 6). TGF-β1, TNF, and BNP were determined by enzyme-linked immunosorbent assay ELISA. Echocardiogram was used to determine left atrial and LV diameters, as well as LV ejection fraction and diastolic function.

**FINDINGS:**

TGF-b1 serum levels were lower in stages B, C, and D, while TNF serum levels were higher in stages C and D of the cardiac form. TGF-β1 presented a weak correlation with LV diastolic function and LV ejection fraction. TNF presented a weak correlation with left atrial and LV diameters and LV ejection fraction.

**CONCLUSIONS:**

TNF is increased, while TGF-β1 is decreased in the cardiac form of chronic Chagas disease. TNF and TGF-β1 serum levels present a weak correlation with LV systolic and diastolic function in Chagas disease patients.

Chagas disease is a neglected tropical disease caused by *Trypanosoma cruzi*, which was originally confined to Latin America. However, the prevalence of Chagas disease outside endemic areas has increased because of intense migrant flow and transmission by alternative routes (Pinto Dias 2013). In Latin America, Chagas disease remains an important health problem, with 6-7 million people estimated to be chronically infected (Dias et al. 2016). The cardiac form of the disease affects 20-30% of chronically infected individuals and presents high morbidity and mortality due to heart failure (HF), sudden death, or stroke (Dias et al. 2016). Therefore, studies of the physiopathogenic aspects of patients who develop Chagas heart disease are extremely important. Changes in the immunologic system are considered one of the main mechanisms of disease progression. While patients with the indeterminate form present an immunologic balance between the host and parasite, patients who progress to the cardiac form present an inflammatory profile (Dutra et al. 2014) with progressive heart tissue damage, cardiac remodelling, and myocardial fibrosis (Dias et al. 2016).

Transforming growth factor β1 (TGF-β1) is a profibrotic protein and multifunctional cytokine that has been implicated in Chagas disease physiopathology (Araujo-Jorge et al. 2012). Studies in animal models suggested that the TGF-β1-pathway is up-regulated during infection (Roman-Campos et al. 2013, Barreto-de-Albuquerque et al. 2015, Ferreira et al. 2016), promoting the intracellular parasite cycle (Waghabi et al. 2005) and inhibiting the immune response against the parasite (Silva et al. 1991), while the TGF-β1 pathway inhibition decreases parasitaemia and cardiac fibrosis and increases survival (de Oliveira et al. 2012). We and others previously showed that TGF-β1 is increased in patients with Chagas disease (Araujo-Jorge et al. 2002, Perez et al. 2011) and has prognostic value (Saraiva et al. 2013). In contrast, others did not demonstrate increased myocardial TGF-β1 mRNA expression (Nogueira et al. 2014) or increased TGF-β1 serum levels in patients with Chagas heart disease (Vilas-Boas et al. 2008).

Tumour necrosis factor (TNF) is a proinflammatory cytokine with increased expression in HF and may play a key role in the immunological imbalance governing Chagas disease progression to the cardiac form (Dutra et al. 2014). Most studies have detected an increase in TNF measured in peripheral blood samples in patients with Chagas heart disease (Ferreira et al. 2003, Lula et al. 2009, Perez et al. 2011, Sousa et al. 2014) and some studies showed an increase in TNF in patients with the indeterminate form (Ferreira et al. 2003, Sousa et al. 2014). However, other studies showed no increase in TNF in the indeterminate form or even in patients with the cardiac form (Pissetti et al. 2009, Keating et al. 2015).

Neurohormone brain natriuretic peptide (BNP) and the N-terminal segment of BNP (pro-BNP), which reflect diastolic pressure inside the left ventricle (LV), are elevated and predict the prognosis of HF (Hartmann et al. 2004). In Chagas disease, pro-BNP and BNP correlates with the LV ejection fraction and diastolic function and left atrial (LA) volume (Barbosa et al. 2007). Moreover, BNP and NTpro-BNP (Sherbuk et al. 2015) are prognostic predictors of Chagas disease.

As both the TGF-β1 and TNF pathways are considered important in Chagas heart disease pathogenesis, we aimed to measure the serum levels of these proteins in non-infected individuals and patients with the indeterminate form or with different stages of the cardiac form. Additionally, we correlated both cytokines levels with LV systolic and diastolic functions and BNP serum values to evaluate their association with worsening cardiac performance.

## SUBJECTS AND METHODS

*Patients* - Patients with the indeterminate or cardiac form of Chagas disease followed at the outpatient service of the Evandro Chagas National Institute of Infectious Diseases were invited to participate in this cross-sectional study. Non-infected subjects were recruited from among those who were referred to our institution to investigate Chagas disease because of a positive epidemiological history but had negative Chagas disease serology. Non-infected subjects with LV systolic dysfunction were excluded. Chagas disease was diagnosed by a positive result in two different serological tests.

All subjects gave written informed consent before their participation. The study was approved by the local ethics committee under number 02826212.6.0000.5262 and conformed to the standards currently applied by the Brazilian National Committee for Research Ethics and Helsinki Declaration of 1975, as revised in 1983.

Patients with any of the following conditions were excluded from the study: previous treatment with benznidazole, co-infectious diseases, pregnancy, auto-immune diseases, cancer, associated cardiovascular diseases that difficult the classification of the cardiac form of Chagas disease, or associated digestive form of Chagas disease.

Patients were classified according to the current Brazilian Chagas disease consensus (Dias et al. 2016) as indeterminate (no evidence of cardiac involvement), stage A (asymptomatic with isolated changes in the electrocardiogram), stage B (asymptomatic with segmental or global LV systolic dysfunction), stage C (symptomatic HF), or stage D (end-stage HF). Race was self-reported.

*TGF-β1, TNF and BNP measurement* - TGF-β1, TNF and BNP measurements were performed by researchers blinded to the clinical classification of the patients. Measurement was performed using commercially available enzyme-linked immunosorbent assay kits (TGF-β1 and TNF: Quantikine ELISA Human ImmunoAssay, R&D Systems, Minneapolis, MN, USA; BNP: BNP-32 human EIA kit, Bachem, Bubendorf, Switzerland) according to the manufacturer’s instructions. For total TGF-β1 dosage, all samples were assayed after acidic pH activation of latent TGF-β1.

*Echocardiography* - Echocardiograms were performed using a phased-array ultrasound system (Vivid 7, GE Medical Systems, Milwaukee, WI) equipped with an M4S phased-array transducer. Cardiac dimensions and Doppler measurements were obtained as previously described (Nascimento et al. 2013). M-mode echocardiography was used to measure LA, LV end-diastolic, and end-systolic diameters. The LV ejection fraction was determined by modified Simpson’s rule with images obtained from apical 4- and 2-chamber views. Pulsed wave Doppler was obtained in the apical 4-chamber view. From transmitral recordings, the peak early (E) and late (A) diastolic filling velocities, E/A ratio, and E-wave deceleration time (DT) were obtained. Peak early (E’) and late (A’) diastolic myocardial velocities are the averages of the values obtained by spectral pulsed tissue Doppler of the mitral annulus at the septal and lateral positions.

*Statistical analysis* - Calculations were conducted using statistical software MedCalc 12.5.0.0. Continuous variables were expressed as the mean ± standard deviation (SD) and discrete variables as percentages. Except for BNP, all studied variables passed standard tests of normality (Kolmogorov-Smirnov test), allowing the use of parametric tests. Data between groups were compared by one-way analysis of variance followed by Student-Newman-Keuls post-hoc analysis. Correlation between echocardiographic parameters and biomarkers was evaluated using Pearson correlation coefficient. The strength of the studied correlations was classified as previously described (Zegers et al. 2010). For statistical analysis, patients in stages C and D were grouped together as both groups were characterised by HF and stage D group had a small sample size. The null hypothesis was rejected at p *<* 0.05.

## RESULTS

*Patient characteristics* - Of the total of 223 recruited subjects, 219 subjects consented to participate in the study. Blood samples were not collected from 14 subjects. Seven non-infected subjects were excluded because of dilated cardiomyopathy. Five patients were excluded because of previous treatment with benznidazole (1), hepatitis C virus coinfection (1), haemolysis of blood sample (1), and associated digestive form (2). The final studied population consisted of 193 subjects: 41 non-infected subjects, 53 patients with Chagas disease and no evidence of cardiac form, and 99 patients with the cardiac form of Chagas disease (24 stage A, 25 stage B, 44 stage C, and 6 stage D).

The mean age of the Chagas disease patients was 57 ± 12 years. Patients with the cardiac form were older than controls or patients with no evidence of the cardiac form. There was no difference in the frequency of racial categories across the studied groups. Co-morbidities were frequently present in patients with Chagas disease. Hypertension was present in 47% of patients, diabetes in 10% of patients, dyslipidaemia in 27% of patients, and coronary artery disease in 2% of patients; 4% of patients were current smokers. There was no significant difference in the frequency of co-morbidities among the studied groups. All patients with stages C or D of the cardiac form used renin-angiotensin system inhibitors, and most also used β-adrenergic blockers, furosemide, and aldosterone inhibitor. Most patients with stage B of the cardiac form also used renin-angiotensin system inhibitors (Table I).

**TABLE I:** Clinical characteristics

	Non-infected subjects (41)	Chagas disease			

		No evidence of cardiac form (53)	Stage A (24)	Stage B (25)	Stages C and D (50)
Age (year)	49 ± 14	52 ± 11	62 ± 11^*†^	60 ± 9^*†^	58 ± 13^*†^
Male (%)	26.8	45.3	33.3	32.0	52
Race (%)					
White	34.1	45.3	58.3	48	50
African-Brazilian	9.7	11.3	12.5	24	18
Brown	51.2	43.4	29.2	28	32
Not determined	4.5	-	-	-	-
Hypertension (%)	48.8	47.2	45.9	60.0	42
Diabetes (%)	17.1	11.3	16.7	0	10
CAD (%)	2.5	1.9	0	4.0	2
Dyslipidemia (%)	24.4	22.6	16.7	44.0	26
Smoking (%)	0	3.8	0	12.0	2
Medication (%)					
ACE inhibitor	-	28	17	56	58
ARB	-	8	29	40	42
Spironolactone	-	0	0	24	78
Carvedilol	-	0	4	56	96
Amiodarone	-	0	8	20	36
Furosemide	-	0	4	28	92
Digoxin	-	0	0	8	42
Warfarin	-	0	8	28	40

ACE: angiotensin-converting enzyme; ARB: angiotensin receptor blocker; CAD: coronary artery disease; *: p < 0.05 vs. non-infected subjects; †: p < 0.05 vs. no evidence of cardiac form; ‡: p < 0.05 vs. stage A.

The place of origin of the patients is described by region and state in Table II. Most patients in all studied groups were born in the Northeast and Southeast regions. There were no significant differences in the region in which patients were born across the studied groups.

**TABLE II:** Geographic origin by region and state

	Non-infected subjects (41)	Chagas disease			

		No evidence of cardiac form (53)	Stage A (24)	Stage B (25)	Stages C and D (50)
Northeast	31 (75.6%)	42 (79.2%)	15 (62.5%)	11 (44%)	35 (70%)
Bahia	18 (44%)	11 (21%)	4 (17%)	4 (16%)	7 (14%)
Paraíba	4 (10%)	10 (19%)	4 (17%)	2 (8%)	8 (16%)
Pernambuco	1 (2%)	3 (6%)	2 (8%)	3 (12%)	7 (14%)
Ceará	4 (10%)	11 (21%)	1 (4%)	1 (4%)	6 (12%)
Alagoas	3 (7%)	6 (11%)	3 (12%)	1 (4%)	4 (8%)
Sergipe	-	1 (2%)	-	-	1 (2%)
Piauí	1 (2%)	-	-	-	2 (4%)
Rio Grande do Norte	-	-	1 (4%)	-	-
Southeast	10 (24.4%)	8 (15.1%)	9 (37.5%)	12 (48%)	12 (24%)
Minas Gerais	6 (14%)	4 (7%)	7 (29%)	11 (44%)	10 (20%)
Rio de Janeiro	4 (10%)	3 (6%)	2 (8%)	1 (4%)	2 (4%)
Espírito Santo	-	1 (2%)	-	-	-
South	0	3 (5.7%)	0	0	0
Rio Grande do Sul	-	2 (4%)	-	-	-
Paraná	-	1 (2%)	-	-	-
North	0	0	0	0	1 (2%)
Pará	-	-	-	-	1(2%)
Midwest	0	0	0	2 (8%)	2 (4%)
Goiás	-	-	-	2 (8%)	2 (4%)

Electrocardiogram findings were more frequent in patients with the cardiac form of Chagas disease, as expected because of Chagas disease classification criteria. Some isolated electrocardiographic changes were not sufficient to indicate that the patient had the cardiac form of disease (Dias et al. 2016). Thus, among patients in the group with no evidence of the cardiac form, two presented left anterior hemiblock and one low QRS voltage. Right bundle branch block, left anterior hemiblock, and primary ST-T wave changes were the most common electrocardiographic changes among patients with the Chagas disease cardiac form. Patients with pacemaker implants were present in all stages of the cardiac form, but most cases were detected in patients with stage C or D of the cardiac form (Table III). Left bundle branch block was also observed in three patients with stage A and two patients with stage B of the cardiac form.

**TABLE III:** Electrocardiographic and echocardiographic characteristics

	Non-infected subjects (41)	Chagas Disease			

		No evidence of cardiac form (53)	Stage A (24)	Stage B (25)	Stages C and D (50)
ECG (%)					
RBBB	12.2	0	62.5^*†^	32.0^†‡^	46.0^*†^
Low QRS voltage	0	1.9	4.2	12.0	20.0^*†‡^
LAHB	4.9	3.8	45.8^*†^	36.0^*†^	46.0^*†^
Electrically inactive area	2.4	0	4.2	16.0^†^	14.0^†^
Primary ST-T wave changes	9.8	0*	29.2^†^	40.0^*†^	48.0^*†^
Pacemaker	0	0	8.3	16.0^*†^	24.0^*†^
Echocardiogram					
LA (mm)	36 ± 4	36 ± 4	39 ± 6^*†^	41 ± 5^*†^	45 ± 5^*†‡#^
LVd (mm)	49 ± 5	50 ± 4	51 ± 3	56 ± 6^*†‡^	68 ± 7^*†‡#^
LVs (mm)	29 ± 4	30 ± 4	31 ± 4	39 ± 8^*†‡^	56 ± 8^*†‡#^
EF (%)	68 ± 7	71 ± 7	69 ± 9	57 ± 14^*†‡^	35 ± 11^*†‡#^
E/A (ratio)	1.4 ± 0.6	1.2 ± 0.5	1.0 ± 0.5	1.0 ± 0.3	2.1 ± 1.3^*†‡#^
DT (ms)	173 ± 42	179 ± 59	190 ± 51	185 ± 78	159 ± 81
E’ (cm/s)	10.7 ± 4.1	9.4 ± 2.8^*^	7.7 ± 2.1^*†^	6.8 ± 2.7^*†^	5.3 ± 1.9^*†‡#^
E/E’	8.8 ± 3.4	8.2 ± 2.6	9.3 ± 3.6	13.0 ± 6.6^*†‡^	18.6 ± 7.9^*†‡#^

A: peak late wave diastolic filling velocity; DT: E-wave deceleration time; E: peak early wave diastolic filling velocity; E’: peak early diastolic mitral annulus velocity; EF: ejection fraction; LA: left atrium; LAHB: left anterior hemi block; LV: left ventricular; LVd: LV end-diastolic diameter; LVs: LV end-systolic diameter; RBBB: right bundle branch block; *: p < 0.05 vs. non-infected subjects rols; †: p < 0.05 vs. no evidence of cardiac form; ‡: p < 0.05 vs. stage A; #: p < 0.05 vs. stage B.

*Echocardiogram findings* - LA and LV diameters were larger and LV ejection fraction was lower in stage B and stages C and D patients than in other groups. LA diameter was also larger in stage A patients than in patients with the indeterminate form or in non-infected subjects. Parameters of LV diastolic function showed progressive worsening of the LV diastolic function across the stages of the cardiac form of Chagas disease. The E’ velocity was lower in stage A patients than in non-infected subjects and patients with no evidence of the cardiac form, and progressively decreased in patients with stage C and D of the cardiac form. In contrast, the E/E’ ratio was higher among patients with stage B than in non-infected subjects, patients with no evidence of the cardiac form, and patients in stage A. The E/E’ ratio further increased in patients in stages C and D of the cardiac form (Table III).

*TGF-β1, TNF and BNP serum values* - TGF-β1 serum values were lower in stages B, C and D of the cardiac form than in non-infected subjects and patients with no evidence of the cardiac form. TNF serum values were higher in patients in stages C and D of the cardiac form than in non-infected subjects and patients with no evidence of the cardiac form.

BNP serum levels were higher in patients who presented HF (stages C and D of the cardiac form) than in all other groups (Table IV).

**TABLE IV:** Serum values of transforming growth factor β1(TGF-β1), tumor necrosis factor (TNF) and brain natriuretic peptide (BNP)

	Non-infected subjects (41)	Chagas disease			

		No evidence of cardiac form (53)	Stage A (24)	Stage B (25)	Stages C and D (50)
TGF-β1 (ng/mL)	48 ± 10	48 ± 11	45 ± 10	42 ± 10^*†^	42 ± 12^*†^
TNF (pg/mL)	3.2 ± 2.1	3.5 ± 4.3	3.9 ± 4.8	3.4 ± 2.9	6.4 ± 7.4^*†^
BNP (pg/mL)	157 ± 123	178 ± 171	240 ± 165	387 ± 492	1224 ± 1615^*†‡#^

*: p < 0.05 vs. non-infected subjects; †: p < 0.05 vs. no evidence of cardiac form; ‡: p < 0.05 vs. stage A; #: p < 0.05 vs. stage B.

**Fig. 1:**
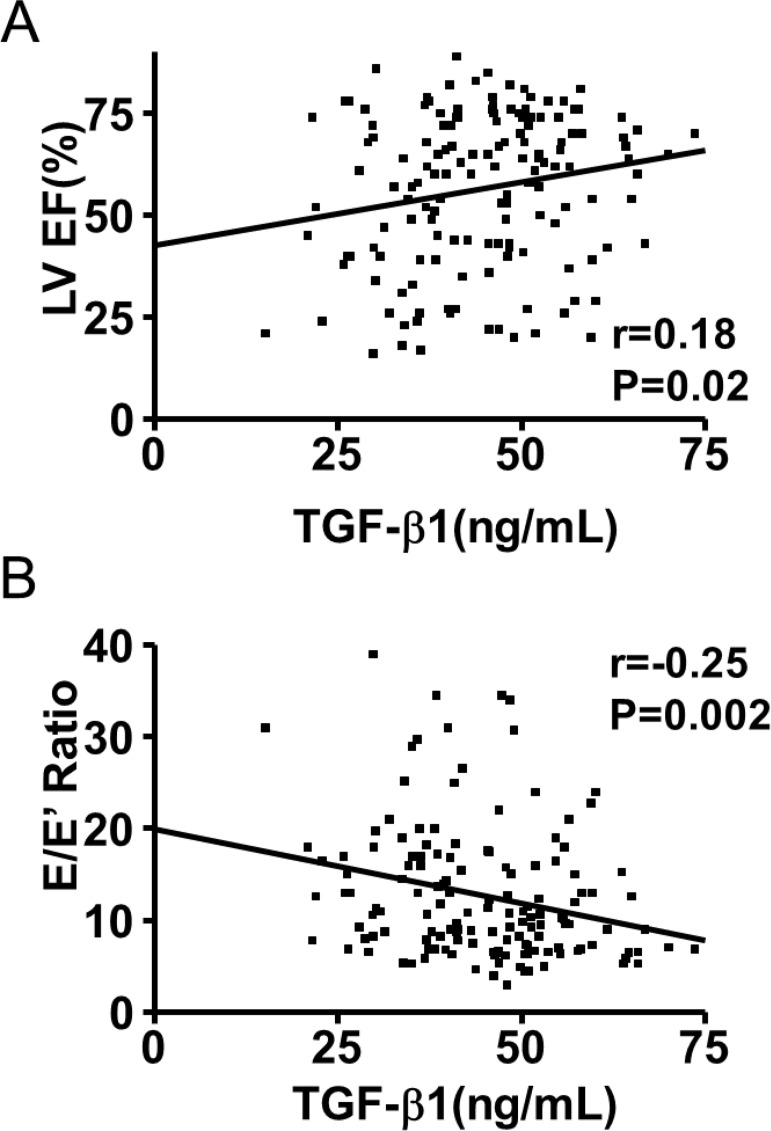
: correlation between transforming growth factor β1 (TGF-β1) serum levels and echocardiographic parameters. TGF-β1 presented a slightly positive correlation with left ventricular ejection fraction (LV EF) (A) and fair negative relationship with peak early diastolic filling and diastolic myocardial velocities (E/E’) ratio (B).

*TGF-β1 and TNF serum levels and LV function* - The correlation between TGF-β1 and TNF serum levels and parameters of LV systolic and diastolic function and BNP serum levels were evaluated only in the population with Chagas disease. TGF-β1 presented a fair negative relationship with E/E’ ratio (r = -0.25; p = 0.002) and slightly positive correlation with LV ejection fraction (r = 0.18; p = 0.02) and fair positive correlation with E’ velocity (r = 0.26; p = 0.002; Fig. 1). TGF-β1 did not show a significant correlation with LA or LV diameters, E/A ratio, DT and BNP serum levels.

**Fig. 2:**
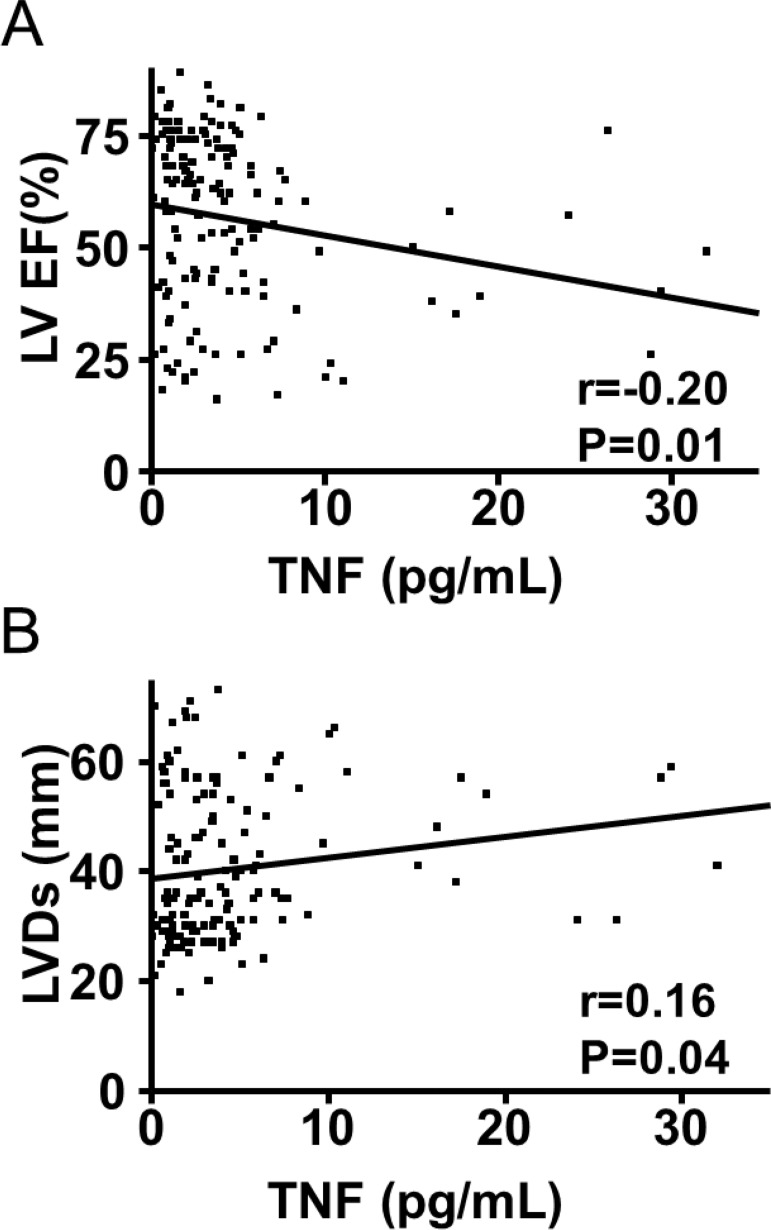
: correlation between tumour necrosis factor (TNF) serum levels and echocardiographic parameters. TNF presented a slightly negative relationship with left ventricular ejection fraction (LV EF) (A) and slightly positive correlation with end-systolic LV diameter (LVDs) (B).

TNF presented a slightly positive relationship with LA (r = 0.16; p = 0.04) and end-systolic LV diameters (r = 0.16; p = 0.04) and slightly negative correlation with LV ejection fraction (r = -0.20; p = 0.01; Fig. 2). TNF was not significantly correlated with any LV diastolic function parameters or BNP serum levels.

We also tested the correlation between TGF-β1 and TNF serum levels and echocardiographic parameters and BNP after excluding all patients with hypertension, diabetes, and any history of coronary artery disease. This comprised a population of 72 patients. In this subpopulation, TGF-β1 still presented a fair positive correlation with E’ velocity (r = 0.36; p = 0.002) and E/A ratio (r = 0.26; p = 0.03), and a nearly significant fair negative correlation with BNP (r = -0.21; p = 0.07). TNF did not present significant correlations with echocardiographic parameters or BNP in this subpopulation.

In contrast, BNP serum levels showed better correlations with echocardiographic parameters of LV diastolic and systolic function than TGF-β1 and TNF. BNP serum levels showed a skewed distribution, and log-transformation was applied before correlation analysis. BNP presented a substantially positive correlation with end-systolic LV diameter (r = 0.62, p < 0.0001) and substantially negative correlation with LV ejection fraction (r = -0.63, p < 0.0001). BNP presented a moderately positive correlation with end-diastolic LV diameter (r = 0.55, p < 0.0001), LA diameter (r = 0.46, p < 0.0001), and E/E’ ratio (r = 0.59, p < 0.0001) and moderately negative correlation with E’ velocity (r = -0.47, p < 0.0001). BNP also presented a fair positive correlation with E/A ratio (r = 0.35, p < 0.0001) and negative correlation with DT (r = -0.21, p = 0.01). We also analysed the correlation between BNP and echocardiographic parameters in the subpopulation after excluding all patients with hypertension, diabetes, and any history of coronary artery disease. BNP still presented a substantial positive correlation with end-systolic LV diameter (r = 0.66, p < 0.0001) and E/E’ ratio (r = 0.65, p < 0.0001) and negative correlation with LV ejection fraction (r = -0.70, p < 0.0001). BNP still presented a moderately positive correlation with end-diastolic LV diameter (r = 0.54, p < 0.0001), LA diameter (r = 0.49, p < 0.0001) and moderately negative correlation with E’ velocity (r = -0.48, p < 0.0001). BNP also still presented a fair positive correlation with E/A ratio (r = 0.38, p = 0.001) and negative correlation with DT (r = -0.38, p = 0.001).

## DISCUSSION

This is the first study to explore the correlation between TGF-β1 and TNF serum levels and both LV systolic and diastolic functions in patients with Chagas disease. Chagas cardiomyopathy is a complex disease that affects approximately 30% of individuals infected with *T. cruzi*. However, previous studies have not reliably predicted which patients will evolve to Chagas cardiomyopathy. Persistence of the parasite within the myocardium and the immune response elicited by such persistence have been proposed to play a key role in Chagas disease progression (Dutra et al. 2014). In contrast, a recent randomised clinical trial known as BENEFIT evaluated the efficacy of benznidazole in modifying the clinical outcomes of patients with chronic Chagas heart disease. A reduction of circulating parasite abundance but a lack of clinical effect on cardiac outcome was observed in a 5-year follow-up study. However, the previous study did not evaluate whether benznidazole treatment modifies progression from the indeterminate to the cardiac form (Morillo et al. 2015). The abundance of inflammatory cytokines may favour Chagas disease progression, while abundance of anti-inflammatory cytokines may favour persistence of the Chagas disease indeterminate form. In this context, several studies demonstrated increased serum levels of inflammatory cytokines among patients with the Chagas disease cardiac form, while other studies revealed an increase in anti-inflammatory cytokines in patients with the indeterminate form (Dutra et al. 2014, Cardillo et al. 2015). Some cytokines may have prognostic value and be correlated with LV function. However, few studies explored the correlation between cytokines serum levels with LV function.

The Chagas disease population followed at our outpatient facility was older compared to those studied previously by our group (Salles et al. 2003). A consequence of this process is the high prevalence of co-morbidities in our studied sample. We did not exclude patients with co-morbidities from our study because we aimed to evaluate cytokine expression and its correlation with LV function in the scenario commonly found in everyday clinical practice. Our echocardiographic data confirmed the worsening of LV systolic and diastolic function according to Chagas disease classification, as we demonstrated previously (Nascimento et al. 2013). The low mean LV ejection fraction, large LV diameters, and high BNP serum levels of patients with stages C and D of the cardiac form reveal the severity of heart disease. BNP serum levels presented significant correlations with all tested echocardiogram parameters.

We found that TNF serum levels were increased only in patients with severe stages of the cardiac form associated with HF. Previous studies found elevated TNF serum levels only in patients with Chagas disease HF (Perez et al. 2011), while others detected elevated TNF serum levels elevated in both indeterminate and Chagas disease HF patients (Ferreira et al. 2003). Other studies (Lula et al. 2009, Sousa et al. 2014) of increased TNF serum levels in patients with the Chagas disease cardiac form did not divide the patients with HF into a separate group for analysis. The number of TNF-producing inflammatory cells within the myocardium is higher in patients with Chagas disease and HF than in patients with the cardiac form and no HF (Rodrigues et al. 2012). TNF showed a positive correlation with LV end-diastolic diameter and negative correlation with LV ejection fraction in patients with Chagas disease (Lula et al. 2009), while others studies found this same correlation only in patients with the cardiac form of Chagas disease (Sousa et al. 2014). TNF was also found to be positively correlated with BNP serum levels (Vilas-Boas et al. 2008, Lula et al. 2009). However, the previous studies did not evaluate the correlation between TNF and LV diastolic function. LV diastolic function is frequently present in Chagas disease and its prevalence increases from the mild to more severe stages of the Chagas disease cardiac form (Nascimento et al. 2013). Moreover, LV diastolic function indices have prognostic value in Chagas disease (Nascimento et al. 2013). Therefore, TNF serum levels may correlate with LV diastolic function. In our study, TNF presented a weakly positive relationship with the LA and end-systolic LV diameters and weakly negative correlation with the LV ejection fraction. These results confirm previous findings showing that TNF is correlated with LV systolic function and add the information that TNF correlates with LA diameter, which is an important index of cardiac function. However, in our study, TNF was not correlated with LV diastolic function or BNP and the correlation with LV diameter or ejection fraction was weaker than that described previously (Lula et al. 2009, Sousa et al. 2014). This may be related to the fact that our patients were older and had a high prevalence of co-morbidities. However, the exclusion of patients with co-morbidities did not increase the strength of the correlation between TGF-β1 or TNF serum levels and BNP or echocardiographic parameters. Another possible reason for the weak correlations between TNF serum levels and echocardiographic parameters is the known effect of cardiovascular drugs used to treat HF on TNF serum levels. Carvedilol (Tatli et al. 2008), spironolactone (Ogino et al. 2014), and angiotensin-converting enzyme inhibitors (Liu & Zhao 1999) decreased TNF serum levels in patients with HF.

Our study revealed a decrease in TGF-β1 serum levels in patients with stages B, C, and D of the cardiac form. We also demonstrated that TGF-β1 had a weak negative relationship with the E/E’ ratio and weak positive correlation with the LV ejection fraction and E’ velocity, indicating that TGF-β1 serum levels decrease with worsening LV systolic and diastolic LV functions. Although we previously found that TGF-β1 was increased in patients with Chagas disease (Araujo-Jorge et al. 2002), we did not confirm this finding in our new patient sample. Other studies also did not detect increased TGF-β1 serum levels in patients with Chagas disease and HF compared to in non-infected subjects (Vilas-Boas et al. 2008). Another study found an increase in TGF-β1 serum levels only in patients with the stage A of the cardiac form (Clark et al. 2015), while TGF-β1 serum levels did not differ between controls and patients with the Chagas disease indeterminate form or more advanced stages of Chagas disease cardiac form. Histopathological studies found no increase in myocardial TGF-β1 mRNA expression in patients with Chagas heart disease compared to in non-infected subjects (Nogueira et al. 2014). Others also found a low prevalence of TGF-β1-positive cells within the myocardium of patients with Chagas disease and HF (Reis et al. 2000). The number of TGF-β-producing inflammatory cells within the myocardium was also found to be similar between patients with Chagas disease cardiac form with or without HF (Rodrigues et al. 2012). One possible explanation for these discrepant results is the different age range of the patients and time living away from endemic areas between studies. The present study included a larger sample of patients than previous studies (Araujo-Jorge et al. 2002, Perez et al. 2011) and patients included in the present study were older and/or lived for a longer time away from endemic areas than those in previous studies (Araujo-Jorge et al. 2002, Perez et al. 2011). Patients who still live in endemic areas or had a lower length of time since their last exposure to new *T. cruzi* infection may show more enhanced activation of the TGF-β1 signalling pathway and different TGF-β1 serum levels than older patients who have lived away from endemic areas for several decades. Moreover, TGF-β1 production depends on the immune response type of each patient, which may be mediated by host genetic factors and medication administered for HF, among other reasons. Regarding medication, patients in the present study were treated with drugs that improved the survival of HF (SOLVD investigators et al. 1991, Packer et al. 1996, Pitt et al. 1999), but decreased TGF-β transcription, such as angiotensin-converting enzyme inhibitor (Kim et al. 1996), spirolactone (Zhou et al. 2016), and carvedilol (Wong et al. 2001). A previous study from our group was performed on blood samples collected between 1998 and 1999 (Araujo-Jorge et al. 2002). At that time, patients were not regularly treated with carvedilol or spironolactone, as trials that demonstrated the beneficial effects of these drugs on HF were published a short time before the study (Packer et al. 1996, Pitt et al. 1999). In the present study, the percentage of patients with HF using carvedilol, angiotensin-converting enzyme inhibitor, and spirolactone was high, similarly to in another study that detected no difference in TGF-β1 serum levels between controls and patients with HF due to Chagas disease (Vilas-Boas et al. 2008).

Therefore, although experimental studies showed that TGF-β1 may have a key role in Chagas disease infection and progression, studies of TGF-β1 serum levels in clinical practice have shown inconsistent results and may not be useful as a surrogate for increased risk of progression or disease severity.

In summary, TNF and TGF-β1 serum levels presented a weak correlation with LV systolic and diastolic functions in patients with Chagas disease. While TNF were increased in patients with Chagas disease HF, TGF-β1 serum levels were decreased in patients with stages B, C, and D of the cardiac form. BNP serum levels were increased only in patients with Chagas disease HF.

## References

[B1] Araujo-Jorge TC, Waghabi MC, Bailly S, Feige JJ (2012). The TGF-beta pathway as an emerging target for Chagas disease therapy. Clin Pharmacol Ther.

[B2] Araujo-Jorge TC, Waghabi MC, Hasslocher-Moreno AM, Xavier SS, Higuchi ML, Keramidas M (2002). Implication of transforming growth factor-beta1 in Chagas disease myocardiopathy. J Infect Dis.

[B3] Barbosa MM, Nunes MC, Ribeiro AL, Barral MM, Rocha MO (2007). N-terminal proBNP levels in patients with Chagas disease: a marker of systolic and diastolic dysfunction of the left ventricle. Eur J Echocardiogr.

[B4] Barreto-de-Albuquerque J, Silva-dos-Santos D, Perez AR, Berbert LR, Santana-van-Vliet E, Farias-de-Oliveira DA (2015). Trypanosoma cruzi infection through the oral route promotes a severe infection in mice: new disease form from an old infection?. PLoS Negl Trop Dis.

[B5] Cardillo F, Pinho RT, Antas PR, Mengel J (2015). Immunity and immune modulation in Trypanosoma cruzi infection. Pathog Dis.

[B6] Clark EH, Marks MA, Gilman RH, Fernández AB, Crawford TC, Samuels AM (2015). Circulating serum markers and QRS scar score in Chagas cardiomyopathy. Am J Trop Med Hyg.

[B7] Oliveira FL, Araujo-Jorge TC, Souza EM, Oliveira GM, Degrave WM, Feige JJ (2012). Oral Administration of GW788388, an inhibitor of transforming growth factor beta signaling, prevents heart fibrosis in Chagas disease. PLoS Negl Trop Dis.

[B8] Dias JC, Ramos AN, Gontijo ED, Luquetti A, Shikanai-Yasuda MA, Coura JR (2016). 2nd Brazilian consensus on Chagas disease, 2015. Rev Soc Bras Med Trop.

[B9] Dutra WO, Menezes CA, Magalhães LM, Gollob KJ (2014). Immunoregulatory networks in human Chagas disease. Parasite Immunol.

[B10] Ferreira RC, Ianni BM, Abel LCJ, Buck P, Mady C, Kalil J (2003). Increased plasma levels of tumor necrosis factor-α in asymptomatic/”indeterminate” and Chagas disease cardiomyopathy patients. Mem Inst Oswaldo Cruz.

[B11] Ferreira RR, Souza EM, Oliveira FL, Ferrao PM, Gomes LH, Mendonça-Lima L (2016). Proteins involved on TGF-β pathway are up-regulated during the acute phase of experimental Chagas disease. Immunobiology.

[B12] Hartmann F, Packer M, Coats AJ, Fowler MB, Krum H, Mohacsi P (2004). Prognostic impact of plasma N-terminal pro-brain natriuretic peptide in severe chronic congestive heart failure: a substudy of the Carvedilol Prospective Randomized Cumulative Survival (COPERNICUS) trial. Circulation.

[B13] Keating SM, Deng X, Fernandes F, Cunha E, Ribeiro AL, Adesina B (2015). Inflammatory and cardiac biomarkers are differentially expressed in clinical stages of Chagas disease. Int J Cardiol.

[B14] Kim S, Ohta K, Hamaguchi A, Yukimura T, Miura K, Iwao H (1996). Effects of an AT1 receptor antagonist, an ACE inhibitor and a calcium channel antagonist on cardiac gene expressions in hypertensive rats. Br J Pharmacol.

[B15] Liu L, Zhao SP (1999). The changes of circulating tumor necrosis factor levels in patients with congestive heart failure influenced by therapy. Int J Cardiol.

[B16] Lula JF, Rocha MO, Nunes MC, Ribeiro AL, Teixeira MM, Bahia MT (2009). Plasma concentrations of tumour necrosis factor-alpha, tumour necrosis factor-related apoptosis-inducing ligand, and FasLigand/CD95L in patients with Chagas cardiomyopathy correlate with left ventricular dysfunction. Eur J Heart Fail.

[B17] Morillo CA, Marin JA, Avezum A, Sosa-Estani S, Rassi A, Rosas F (2015). Randomized trial of benznidazole for chronic Chagas’ cardiomyopathy. N Engl J Med.

[B18] Nascimento CA, Gomes VA, Silva SK, Santos CR, Chambela MC, Madeira FS (2013). Left atrial and left ventricular diastolic function in chronic chagas disease. J Am Soc Echocardiogr.

[B19] Nogueira LG, Santos RH, Fiorelli AI, Mairena EC, Benvenuti LA, Bocchi EA (2014). Myocardial gene expression of T-bet, GATA-3, Ror-gammat, FoxP3, and hallmark cytokines in chronic Chagas disease cardiomyopathy: an essentially unopposed TH1-type response. Mediators Inflamm.

[B20] Ogino K, Kinugasa Y, Kato M, Yamamoto K, Hisatome I, Anker SD (2014). Spironolactone, not furosemide, improved insulin resistance in patients with chronic heart failure. Int J Cardiol.

[B21] Packer M, Bristow MR, Cohn JN, Colucci WS, Fowler MB, Gilbert EM (1996). The effect of carvedilol on morbidity and mortality in patients with chronic heart failure. U.S. Carvedilol Heart Failure Study Group. N Engl J Med.

[B22] Perez AR, Silva-Barbosa SD, Berbert LR, Revelli S, Beloscar J, Savino W (2011). Immunoneuroendocrine alterations in patients with progressive forms of chronic Chagas disease. J Neuroimmunol.

[B23] Pinto Dias JCP (2013). Human chagas disease and migration in the context of globalization: some particular aspects. J Trop Med.

[B24] Pissetti CW, Correia D, Braga T, Faria GE, Oliveira RF, Ribeiro BM (2009). Association between the plasma levels of TNF-alpha, IFN-gamma, IL-10, nitric oxide and specific IgG isotypes in the clinical forms of chronic Chagas disease. Rev Soc Bras Med Trop.

[B25] Pitt B, Zannad F, Remme WJ, Cody R, Castaigne A, Perez A (1999). The effect of spironolactone on morbidity and mortality in patients with severe heart failure. Randomized Aldactone Evaluation Study Investigators. N Engl J Med.

[B26] Reis MM, Higuchi ML, Aiello VD, Benvenuti LA (2000). Growth factors in the myocardium of patients with chronic chagasic cardiomyopathy. Rev Soc Bras Med Trop.

[B27] Rodrigues DBR, Reis MA, Romano A, Pereira SA, Teixeira VP, Tostes S (2012). In situ expression of regulatory cytokines by heart inflammatory cells in Chagas’ disease patients with heart failure. Clin Dev Immunol.

[B28] Roman-Campos D, Sales P, Duarte HL, Gomes ER, Lara A, Campos P (2013). Novel insights into the development of chagasic cardiomyopathy: role of PI3Kinase/NO axis. Int J Cardiol.

[B29] Salles G, Xavier S, Sousa A, Hasslocher-Moreno A, Cardoso C (2003). Prognostic value of QT interval parameters for mortality risk stratification in Chagas’ disease: results of a long-term follow-up study. Circulation.

[B30] Saraiva RM, Waghabi MC, Vilela MF, Madeira FS, Silva GM, Xavier SS (2013). Predictive value of transforming growth factor-beta1in Chagas disease: towards a biomarker surrogate of clinical outcome. Trans R Soc Trop Med Hyg.

[B31] Sherbuk JE, Okamoto EE, Marks MA, Fortuny E, Clark EH, Galdos-Cardenas G (2015). Biomarkers and mortality in severe Chagas cardiomyopathy. Glob Heart.

[B32] Silva JS, Twardzik DR, Reed SG (1991). Regulation of Trypanosoma cruzi infections in vitro and in vivo by transforming growth factor beta (TGF-beta). J Exp Med.

[B33] SOLVD Investigators, Yusuf S, Pitt B, Davis CE, Hood WB, Cohn JN (1991). Effect of enalapril on survival in patients with reduced left ventricular ejection fractions and congestive heart failure. The SOLVD Investigators. N Engl J Med.

[B34] Sousa GR, Gomes JA, Fares RC, Damasio MP, Chaves AT, Ferreira KS (2014). Plasma cytokine expression is associated with cardiac morbidity in chagas disease. PLoS ONE.

[B35] Tatli E, Kurum T, Aktoz M, Buyuklu M (2008). Effects of carvedilol on right ventricular ejection fraction and cytokines levels in patients with systolic heart failure. Int J Cardiol.

[B36] Vilas-Boas F, Feitosa GS, Soares MB, Pinho JA, Nascimento T, Barojas MM (2008). Invasive and noninvasive correlations of B-type natriuretic peptide in patients with heart failure due to Chagas cardiomyopathy. Congest Heart Fail.

[B37] Waghabi MC, Keramidas M, Bailly S, Degrave W, Mendonça-Lima L, Soeiro MN (2005). Uptake of host cell transforming growth factor-beta by Trypanosoma cruzi amastigotes in cardiomyocytes: potential role in parasite cycle completion. Am J Pathol.

[B38] Wong VY, Laping NJ, Nelson AH, Contino LC, Olson BA, Gygielko E (2001). Renoprotective effects of carvedilol in hypertensive-stroke prone rats may involve inhibition of TGF beta expression. Br J Pharmacol.

[B39] Zegers M, Bruijne MC, Wagner C, Groenewegen PP, van der WG, Vet HC (2010). The inter-rater agreement of retrospective assessments of adverse events does not improve with two reviewers per patient record. J Clin Epidemiol.

[B40] Zhou H, Xi D, Liu J, Zhao J, Chen S, Guo Z (2016). Spirolactone provides protection from renal fibrosis by inhibiting the endothelial-mesenchymal transition in isoprenaline-induced heart failure in rats. Drug Des Devel Ther.

